# Factors related to self-medication in undergraduate nursing students: a scoping review

**DOI:** 10.17533/udea.iee.v43n2e07

**Published:** 2025-07-25

**Authors:** Hemelly Nogueira Guimarães Silveira, Silvia Regina Secoli, Thaís São-João, Aline Carrilho Menezes, Flávia de Oliveira, Silmara Nunes Andrade, Danilo Donizetti Trevisan

**Affiliations:** 1 Nurse, Master student. Email: hemelly98@gmail.com. https://orcid.org/0000-0002-3807-1475 Universidade Federal de São João del-Rei Brazil hemelly98@gmail.com; 2 Nurse, Ph.D. Email: secolisi@usp.br. https://orcid.org/0000-0003-4135-6241 Universidade de São Paulo Brazil secolisi@usp.br; 3 Nurse, Ph.D. Email: thais_sao-joao@uri.edu. https://orcid.org/0000-0002-8520-6483 University of Rhode Island USA thais_sao-joao@uri.edu; 4 Nurse, Ph.D. Email: alinecarrilhomenezes@gmail.com. https://orcid.org/0000-0001-7658-4039 Universidade Federal de São João del-Rei Brazil alinecarrilhomenezes@gmail.com; 5 Nurse, Ph.D. Email: flaviadeoliveira@ufsj.edu.br. https://orcid.org/0000-0002-9044-6588 Universidade Federal de São João del-Rei Brazil flaviadeoliveira@ufsj.edu.br; 6 Nurse, Ph.D. Email: silmaranunesandrade@ufsj.edu.br. https://orcid.org/0000-0002-1975-0827 Universidade Federal de São João del-Rei Brazil silmaranunesandrade@ufsj.edu.br; 7 Nurse, Ph.D. Email: ddtrevisan@ufsj.edu.br. Corresponding author. https://orcid.org/0000-0002-6998-9166 Universidade Federal de São João del-Rei Brazil ddtrevisan@ufsj.edu.br; 8 Federal University of São João del Rei (UFSJ), Divinópolis/MG, Brazil Universidade Federal de São João del-Rei Federal University of São João del Rei Divinópolis MG Brazil; 9 University of São Paulo (USP), São Paulo/SP, Brazil Universidade de São Paulo University of São Paulo (USP) São Paulo SP Brazil; 10 University of Rhode Island (URI), Rhode Island/RI, United State University of Rhode Island University of Rhode Island (URI) Rhode Island RI USA

**Keywords:** self-medication, drug utilization, drug-related side effects and adverse reactions, students, nursing, nursing, automedicación, utilización de medicamentos, efectos colaterales y reacciones adversas relacionados con medicamentos, estudiantes de enfermería, enfermería., automedicação, uso de medicamentos, efeitos colaterais e reações adversas relacionados a medicamentos, estudantes de enfermagem, enfermagem

## Abstract

**Objective:**

The aim of this study was to map and summarize the extent and type of evidence related to self-medication among undergraduate nursing students.

**Method:**

A scoping review was conducted in accordance with the guidelines of the Joanna Briggs Institute. The guiding questions of the interviews were the following: 1) "What are the reasons/motivations and health conditions leading to self-medication behavior in nursing students?"; 2) "What are the sources of information involved in the decision-making process for self-medication?"; 3) "What medications are used in self-medication?"; 4) "What are the perceptions of risks and benefits of this behavior?"; 5) "What are the knowledge, beliefs/attitudes, and practices regarding self-medication?" The descriptors used were: "self-medication" AND "students, nursing" combined with their synonyms. A total of 55 articles were included from searches in the MEDLINE, Web of Science, Scopus, and Virtual Health Library databases, including gray literature.

**Results:**

The analysis of the studies resulted in ten categories characterizing the factors of self-medication: clinical conditions (*n*=52), reasons/motives (*n*=54), recall period (*n*=22), drug classes (*n*=54), sources of advice (n=53), perception of risks (*n*=47) and benefits (n=21), knowledge (*n*=27), beliefs and attitudes (*n*=27), and practices (*n*=20).

**Conclusion:**

This study showed that clinical, socioeconomic, and behavioral factors are common themes described in the literature on self-medication among undergraduate nursing students. There are opportunities, especially in training, to improve self-medication practices and patient safety among future nurses.

## Resumen

**Objetivo.:**

El objetivo de este estudio fue mapear y sintetizar el alcance de la evidencia relacionada con la automedicación en estudiantes universitarios de enfermería.

**Métodos.:**

Se llevó a cabo una revisión de alcance de acuerdo con las directrices del Instituto Joanna Briggs. Las preguntas guía fueron: 1) «¿cuáles son las razones o motivos y las situaciones de salud que conducen al comportamiento de automedicación en estudiantes de enfermería?»; 2) «¿cuáles son las fuentes de información implicadas en la decisión del comportamiento de automedicación?»; 3) «¿cuáles son los medicamentos utilizados en la automedicación?»; 4) ¿cuáles son las percepciones sobre los riesgos y beneficios de este comportamiento?»; 5) «¿cuáles son los conocimientos, creencias, actitudes y prácticas que implican la automedicación?». Para la estrategia de búsqueda se utilizaron los descriptores «automedicación» Y «estudiantes, enfermería» con sus respectivos sinónimos. Se incluyeron 55 artículos procedentes de la búsqueda en las bases de datos MEDLINE, Web of Science, Scopus, Virtual Health Library; y se revisó literatura gris.

**Resultados.:**

El análisis de los estudios dio como resultado diez categorías que caracterizan los factores de la automedicación: condiciones clínicas (*n*=52), razones o /motivos (*n*=54), periodo de recuerdo (*n*=22), clases de fármacos (*n*=54), fuentes de consejo (*n*=53), percepción de riesgos (*n*=47) y beneficios (*n*=21), conocimientos (*n*=27), creencias y actitudes (*n*=27) y prácticas (*n*=20).

**Conclusión.:**

Este estudio mostró que los factores clínicos, socioeconómicos y conductuales son temas comunes descritos en la bibliografía en relación con la automedicación entre los estudiantes universitarios de enfermería. Existen oportunidades, especialmente de carácter formativo, para mejorar la práctica de la automedicación entre los futuros enfermeros.

## Resumo

**Objetivo:**

Objetivou-se mapear e sintetizar a extensão de evidência relacionada à automedicação em estudantes de graduação em Enfermagem.

**Método:**

Revisão de escopo realizada conforme diretrizes do Instituto *Joanna Briggs*. As questões norteadoras foram: 1) “quais são as razões/motivos e condições de saúde que levam ao comportamento da automedicação em estudantes de enfermagem? ”; 2) “quais são as fontes de informação envolvidas na decisão do comportamento da automedicação?”; 3) “quais são os medicamentos utilizados na modalidade de automedicação?”; 4) “quais são as percepções acerca de riscos e benefícios deste comportamento?”; 5) “quais são os conhecimentos, as crenças/atitudes e as práticas envolvendo a automedicação?” Os descritores “s*elf medication*” AND “*students, nursing”* foram utilizados com seus respectivos sinônimos para a estratégia de busca. Foram incluídos 55 artigos a partir da busca em bases de dados MEDLINE, *Web of Science*, *Scopus*, Biblioteca Virtual em Saúde; incluindo a literatura cinzenta.

**Resultados:**

Da análise dos estudos, resultaram dez categorias que caracterizam os fatores da automedicação: condições clínicas (*n*=52), razões/motivos (*n*=54), período recordatório (*n*=22), classes de medicamentos (*n*=54), fontes de aconselhamento (*n*=53), percepção de riscos (*n*=47) e benefícios (*n*=21), conhecimento (*n*=27), crenças e atitudes (*n*=27) e práticas (*n*=20).

**Conclusão:**

Este estudo evidenciou que fatores natureza clínica, socioeconômica e comportamental foram temas comuns descritos na literatura acerca automedicação em estudantes de graduação em Enfermagem. Existem oportunidades, sobretudo de caráter formativo, para melhorar a pratica da automedicação dos futuros enfermeiros.

## Introduction

Epidemiological evidence reports that self-medication is a global, growing phenomenon, highly prevalent across different age groups.^1^^,^^2^ However, university students appear to be more prone to practicing self-medication. A systematic review with meta-analysis, covering 60,938 university students, revealed a global prevalence of self-medication at 70.1%. Among health science students, the prevalence was higher (97.2%).^3^ The main motivations for self-medication among university students, including nursing students, involve various factors such as high educational level, easy access to medications, quick symptom resolution, limited time to seek healthcare services, high consultation and examination costs, and difficulty accessing healthcare services.^4^^-^^8^ Nevertheless, although self-medication may seem like a practical and quick self-care strategy, from a pharmacovigilance perspective, it can become a public health issue.^9^ The negative effects of this practice include the risk of adverse drug events (ADEs), drug interactions, antimicrobial resistance (AMR), and increased healthcare costs.^10^

In this context, where evidence on the concept of self-medication^10^ and its prevalence^3^ has contributed to a broader understanding of the issue, knowledge gaps still need to be addressed to support efforts to tackle this public health problem, especially among undergraduate nursing students. 

In this group, self-medication may indirectly affect future practices related to medication management and significantly impact patient safety. Nurses play a crucial role in the medication administration process and, in multiprofessional collaboration, engage in various activities related to medication safety.^11^ Thus, this scoping review aimed to map and synthesize the available evidence on self-medication among undergraduate nursing students. 

## Method

Study design. This scoping review followed the recommendations of the Joanna Briggs Institute (JBI) - Methodology for JBI Scoping Review^12^ and the Preferred Reporting Items for Systematic Reviews and Meta-Analyses extension for Scoping Reviews (PRISMA-ScR*).*^13^ The review protocol was registered in the Open Science Framework - https://doi.org/10.17605/OSF.IO/ZYFG9. The guiding questions of the interviews were the following: *1) "What are the reasons/motivations and health conditions leading to self-medication behavior in nursing students?"; 2) "What are the sources of information involved in the decision-making process for self-medication?"; 3) "What medications are used in self-medication?"; 4) "What are the perceptions of risks and benefits of this behavior?"; 5) "What are the knowledge, beliefs/attitudes, and practices regarding self-medication?"* The following steps were undertaken: defining and aligning the objectives and research question; setting inclusion criteria; planning and conducting the search strategy; selecting studies; extracting evidence; analyzing evidence; and summarizing the results. The research question was formulated using the *participants, concept, and context* (PCC) strategy: P (Participants) - undergraduate nursing students; C (Concept) - aspects related to self-medication; C (Context) - public/private higher education institutions worldwide. 

Inclusion and exclusion criteria. Scientific studies addressing self-medication among undergraduate nursing students were included. Primary studies and systematic reviews were assessed without temporal or language restrictions. Duplicates, studies without full text, and conference abstracts were excluded.

Data sources and search strategies. Data collection occurred in June 2024 from the following databases: Medical Literature Analysis and Retrieval System Online (MEDLINE/via PubMed), Web of Science Core Collection/Clarivate Analytics (WoS), Scopus (Elsevier), and *Biblioteca Virtual em Saúde* (BVS). Subsequently, the search was expanded to include grey literature from sources such as: *Portal de Teses e Dissertações da Coordenação de Aperfeiçoamento de Pessoal do Nível Superior* (CAPES), Europe E-Theses Portal (DART), Electronic Theses Online Service (EThOS), *Repositório Científico de Acesso Aberto de Portugal* (RCAAP), National ETD Portal, Theses Canada, *Portal de Tesis Latinoamericanas* e Open Grey. The research team defined the search strategy using Health Sciences Descriptors (DeCS) and/or Medical Subject Headings (MeSH). Boolean operators AND and/or OR were employed according to the specifics of each database. The English descriptors used were: "*self medication*" AND "*students, nursing*" combined with their synonyms. Table 1 presents the detailed search strategy.

**Table 1:** Terms and search strategies applied to each database

Database	Search strategy	Results
MEDLINE via PUBMED	#1 (("*Self Medication"[MeSH Terms] OR ("self"[All Fields] AND "medication"[All Fields]) OR "Self Medication"[All Fields] OR ("medication"[All Fields] AND "self"[All Fields]) OR "medication self"[All Fields]) AND ("Self Medication"[MeSH Terms] OR ("self"[All Fields] AND "medication"[All Fields]) OR "Self Medication"[All Fields] OR ("medications"[All Fields] AND "self"[All Fields]) OR "medications self"[All Fields]) AND ("Self Medication"[MeSH Terms] OR ("self"[All Fields] AND "medication"[All Fields]) OR "Self Medication"[All Fields] OR ("self"[All Fields] AND "medications"[All Fields]) OR "self medications"[All Fields])* = 39254 #2 ("*students, nursing"[MeSH Terms] OR ("students"[All Fields] AND "nursing"[All Fields]) OR "nursing students"[All Fields] OR ("pupil"[All Fields] AND "nurses"[All Fields]) OR "pupil nurses"[All Fields]) AND ("students, nursing"[MeSH Terms] OR ("students"[All Fields] AND "nursing"[All Fields]) OR "nursing students"[All Fields] OR ("student"[All Fields] AND "nursing"[All Fields]) OR "student nursing"[All Fields]) AND ("students, nursing"[MeSH Terms] OR ("students"[All Fields] AND "nursing"[All Fields]) OR "nursing students"[All Fields] OR ("nurses"[All Fields] AND "pupil"[All Fields])) AND ("students, nursing"[MeSH Terms] OR ("students"[All Fields] AND "nursing"[All Fields]) OR "nursing students"[All Fields] OR ("nurse"[All Fields] AND "pupil"[All Fields]) OR "nurse pupil"[All Fields]) AND ("students, nursing"[MeSH Terms] OR ("students"[All Fields] AND "nursing"[All Fields]) OR "nursing students"[All Fields] OR ("pupil"[All Fields] AND "nurse"[All Fields]) OR "pupil nurse"[All Fields]) AND ("students, nursing"[MeSH Terms] OR ("students"[All Fields] AND "nursing"[All Fields]) OR "nursing students"[All Fields] OR ("nursing"[All Fields] AND "student"[All Fields]) OR "nursing student"[All Fields]) AND ("students, nursing"[MeSH Terms] OR ("students"[All Fields] AND "nursing"[All Fields]) OR "nursing students"[All Fields] OR ("nursing"[All Fields] AND "students"[All Fields]))* = 61158 #3 #1 and #2 = 252	252
*Scopus*	#1 ( *TITLE-ABS-KEY ( self AND medication ) OR TITLE-ABS-KEY ( medication, AND self ) OR TITLE-ABS-KEY ( medications, AND self ) OR TITLE-ABS-KEY ( self AND medications ) )* = 64939 #2 *( TITLE-ABS-KEY ( students, AND nursing ) OR TITLE-ABS-KEY ( student, AND nursing ) OR TITLE-ABS-KEY ( nursing AND student ) OR TITLE-ABS-KEY ( nursing AND students* ) = 70952 #3 #1 AND #2 = 305	305
*Web of Science*	#*1 ALL=(Self Medication OR Self Medications OR Medication, Self OR Medications, Self OR Self-Medication OR Self-Medications OR "Self Medication" OR "Self Medications" OR "Medication, Self" OR "Medications, Self" OR "Self-Medication" OR "Self-Medications" )* = 45406 #2 *ALL=(Students, Nursing OR Student, Nursing OR Nursing Student OR Nursing Students OR "Students, Nursing" OR "Student, Nursing" OR "Nursing Student" OR "Nursing Students")* = 64603 #3 #1 and #2 = 435	435
BVS	"*Self Medication*" AND "*Students, Nursing*"	34

Study selection and data extraction. Study selection occurred in three consecutive stages by two independent reviewers: 1. title reading, which had to include the terms self-medication and/or university students or undergraduate nursing students; 2. abstract reading; and 3. full-text reading. Discrepancies or doubts were resolved by consensus with a third independent reviewer. For data extraction, a spreadsheet was created with sections for authorship, title, journal, year and country of publication, objectives, study type, recall period for self-medication, health conditions, drug classes according to the *Anatomical Therapeutic Chemical (ATC) classification,*^14^ reasons/motivations for self-medication, sources of advice, perception of risks and benefits, knowledge, attitudes, and practices regarding self-medication. 

Data analysis and treatment. In each publication, the central elements related to the problem were identified and extracted through full-text reading. The selected studies were characterized and then organized into thematic categories. Descriptive statistics were used to present absolute and relative frequencies. The research team participated in interpreting and synthesizing the data from the articles through narrative discussion, which enabled the association of tabulated results with the research objectives and guiding questions.

## Results

**Figure 1:**
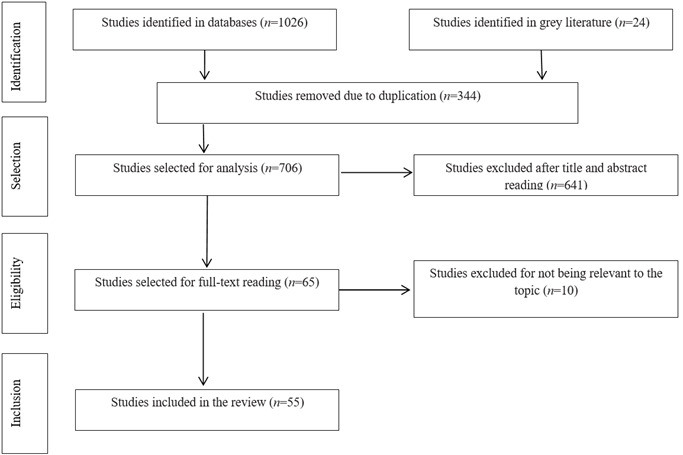
Flowchart of the study selection process according to PRISMA ScR recommendations.

Of the 65 studies selected for full-text reading, 10 were excluded for reasons described in the PRISMA-ScR flowchart.^13^ The sample consisted of 55 studies (Figure 1).

Table 2 presents the authorship, title, year, country of publication, study type, and journal of each included study. The studies were conducted in 23 different countries, with nearly half (47.3%) originating from Asia and about one-third (29.1%) from South America. The publication period spanned from 2004 to 2024, with nearly half (49.1%) published between 2020 and 2024. Most studies (69.1%) were published in English. Almost all studies (90.9%) used a quantitative methodological approach; there was a wide variety of journals publishing on the topic (Table 2). Ten categories were identified to synthesize the factors involved in self-medication among undergraduate nursing students, which are described below and detailed in Table 3. 

*Clinical Conditions:* A total of 94.5% of the studies identified the clinical conditions that motivated nursing students to practice self-medication. Among these, all highlighted "general pain," and a significant portion (80.7%) pointed to symptoms related to upper respiratory tract infections. 

*Reasons or motivations for self-medication:* All studies described the reasons or motivations for self-medication, with predominant aspects related to limited access to healthcare services, including financial/economic factors (77.7%). 

*Medications used:* All studies examined the medications used in self-medication. Among these, the prevalent groups according to *Anatomical Therapeutic Chemical Classification* included antibacterials or antifungals - Class J (61.1%), analgesics, opioids, and antipyretics - Class N (61.1%), and non-steroidal anti-inflammatory drugs. 

*Recall period:* Less than half of the studies (40.0%) indicated the recall period. Of these, 54.6% reported self-medication use in the last four to 12 months, and ten studies (45.4%) reported a period of 15 days to three months. 

*Sources of advice:* Regarding sources of advice, almost all studies (96.4%) noted this variable. Pharmacy attendants or pharmacists were the most self-reported for assistance (69.8%), followed by fellow students (58.5%) and family members (54.7%). 

*Perception of risks:* Concerning risk perceptions of self-medication, described in 85.4% of the studies, participants most frequently listed the risk of adverse reactions and side effects (51.1%), the possibility of masking or delaying appropriate treatments and diagnoses (51.1%), and the possibility of drug interactions (34.0%). 

*Perception of benefits:* More than one-third of the studies (38.2%) reported the benefits of the practice, predominantly including the promotion of self-care (80.9%). 

*Knowledge (n = 27):* Regarding knowledge of self-medication, about half of the articles (49.1%) provided information, showing that students considered their pharmacology knowledge insufficient to promote safe or effective self-medication (37.0%) or sufficient only for certain drug classes (37.0%).

*Beliefs and Attitudes:* Beliefs/attitudes were addressed in one-third of the studies (30.9%). Findings indicated that nursing students were favorable to advising on self-medication (29.4%) and endorsed this practice among their peers. 

*Practices:* Regarding daily self-medication practices, reported in 36.4% of the studies, frequent use of medications was observed in 55.0%, and the combination of different medications was reported in four articles (20.0%).

**Table 2:** Characterization of studies according to year, authorship, periodical, title and country

Year	Authorship	Journal	Title	Country
2024	Batalla, A. *et al.*^15^	Healthcare (Basel)	Dermatology Self-Medication in Nursing Students and Professionals: A Multicentre Study	Spain
2023	Nakato, G. *et al.*^16^	Plos One	Practices and drivers of self-medication with antibiotics among undergraduate medical students in Eastern Uganda: A cross-sectional study	Uganda
	Malli, IA. *et al.*^17^	Preventive Medicine Reports	The prevalence of self-medication and its associated factors among college students: Cross-sectional study from Saudi Arabia	Saudi Arabia
	Guevara-Alburqueque, MA. *et al.*^18^	Revista Cubana de Farmácia	Self-medication during COVID-19 in Nursing Students at a Peruvian University	Peru
	El-Kader, RGA. *et al.*^19^	Health Science Reports	Assessment of health-related behaviors among medical students: A cross-sectional study	United Arab Emirates
	Shanmugam, AJ. *et al.*^20^	International Journal of Electrical and Electronics Engineering	Analysis of Undergraduate Student's Knowledge of Self-Medication Practice using Machine Learning Algorithms	Saudi Arabia
	Zambrano Barriga, F.P. *et al.*^21^	Salud, Ciencia y Tecnologia	Factors causing self-medication in nursing students	Equator
2022	Aranha, PR. *et al.*^12^	Journal of Health and Allied Sciences NU	Assessment of Health-Seeking Behavior among Undergraduate Students at a University	India
	Manikanta, KN. *et al.*^23^	Journal of Pharmaceutical Negative Results	Antibiotic Use and Resistance: A Cross-Sectional Study Exploring Knowledge, Attitudes and Practices among Medical, Dental, Pharmacy and Nursing Students	India
	Janatolmakan, M. *et al.*^4^	Nursing Research and Practice	The Reasons for Self-Medication from the Perspective of Iranian Nursing Students: A Qualitative Study	Iran
	Castro-Cataño, ME. *et al.*^24^	Enfermería Global	Self-medication among undergraduate nursing students	Colombia
	Terzic, D. *et al.*^5^	Serbian Journal of Experimental and Clinical Research	Self-medication with antibiotics among nursing students in Serbia: pilot study	Serbia
2021	Fetensa, G. *et al.*^25^	Journal of Pharmaceutical Policy and Practice	Prevalence and predictors of self-medication among university students in Ethiopia: a systematic review and meta-analysis	Ethiopia
	Saddique, N. *et al.*^6^	Pakistan Journal of Medical & Health Sciences	Prevalence, Awareness Level and Attitude of Self-Medication Among Student Nurses	Pakistan
	Akande-Sholabi, W. *et al.*^26^	Journal of Pharmaceutical Policy and Practice	Prevalence, knowledge and perception of self-medication practice among undergraduate healthcare students	Nigeria
	Faqihi, AHMA. *et al.*^27^	Annales Pharmaceutiques Françaises	Self-medication practice with analgesics (NSAIDs and acetaminophen), and antibiotics among nursing undergraduates in University College Farasan Campus, Jazan University, KSA	United States
	González-Muñoz, F. *et al.*^28^	Educación Médica	Self-medication among final year students of Nursing, Physiotherapy and Medicine at the University of Cordoba	Spain
	Bharati, JP. *et al.*^29^	Journal of Nepal Medical Association	Self-medication in primary dysmenorrhea among medical and nursing undergraduate students of a tertiary care hospital: A descriptive cross-sectional study	Nepal
	Andrés, MIG. *et al.*^30^	International Journal of Environmental Research and Public Health	Self-medication of drugs in nursing students from Castile and Leon (Spain)	Spain
	Naseef, H. *et al.*^7^	Journal of Young Pharmacists	Pattern Knowledge and Determinants of Analgesic Self-medication among Undergraduate Students in the Faculty of Pharmacy, Nursing and Health Professions	Palestine
	Kifle, ZD. *et al.*^9^	Inquiry: The Journal of Health Care Organization, Provision, and Financing	Self-medication Practice and Associated Factors among Private Health Sciences Students in Gondar Town, North West Ethiopia. A Cross-sectional Study	Ethiopia
	Araújo Júnior, AG. *et al.*^31^	Arquivos em Odontologia	Prevalência da automedicação em acadêmicos de odontologia e enfermagem em uma instituição pública brasileira	Brazil
2020	Bohomol, E. *et al.*^32^	Ciência, Cuidado e Saúde	Prática da automedicação entre estudantes de enfermagem de instituição de ensino superior	Brazil
	Parra-Fernández ML. *et al.*^33^	International Journal of Environmental Research and Public Health	Management of Primary Dysmenorrhea among University Students in the South of Spain and Family Influence	Spain
	Khatony, A. *et al.*^34^	BMC Nursing	Nursing students' perceived consequences of self-medication: A qualitative study	Iran
	Chindhalore, C. *et al.*^35^	Journal of Education and Health Promotion	Comparison of self-medication practices with analgesics among undergraduate medical and paramedical students of a tertiary care teaching institute in Central India - A questionnaire-based study	India
	Olorunfemi, O. *et al.*^36^	Journal of Integrative Nursing	Assessing the reasons for increase in self.medication and control measures among student nurses in University of Benin Teaching Hospital, Edo State, Nigeria	Nigeria
	Sharma, K. *et al.*^37^	Clinical Epidemiology and Global Health	Self-medication practices with antibiotics among nursing students: A cross-sectional descriptive survey at tertiary care teaching hospital in Uttarakhand	India
2019	Nogueira, WB. *et al.*^38^	Revista de Enfermagem UFPE on-line	Automedicação: prática entre graduandos de enfermagem	Brazil
	Al Essa, M. *et al.*^39^	Saudi Pharmaceutical Journal	Practices, awareness and attitudes toward self-medication of analgesics among health sciences students in Riyadh, Saudi Arabia	Saudi Arabia
	Karaman, A. *et al.*^40^	Florence Nightingale Journal of Nursing	Rational Drug Usage Status of Nursing Students	Turkey
	Colares, KTP. *et al.*^41^	Revista de Enfermagem UFPE online	Prevalência e fatores associados à automedicação em acadêmicos de enfermagem	Brazil
2018	Anand, S. *et al.*^42^	Asian Journal of Pharmaceutical and Clinical Research	A study of pattern of self-medication among students for dysmenorrhea	India
	Abdi, A. *et al.*^43^	BMC Pharmacology and Toxicology	Prevalence of self-medication practice among health sciences students in Kermanshah, Iran	Iran
	Donmez, S. *et al.*^44^	International Journal of Pharmacology	Knowledge, attitude and practice of self-medication with antibiotics among nursing students	Turkey
	Kim, Hae-Ok. *et al.*^45^	Health Communication	Knowledge and Attitude about Drugs and the Current Status of Self-medication of Nursing Students	South Korea
	Esan, DT. *et al.*^46^	Journal of Environmental and Public Health	Assessment of Self-Medication Practices and Its Associated Factors among Undergraduates of a Private University in Nigeria	Nigeria
2017	Sajith, M. *et al.*^47^	The Open Public Health Journal	Self-medication practices among health care professional students in a tertiary care hospital, Pune	India
	Tse, MMY. *et al.*^48^	Cyberpsychology, Behavior and Social Networking	Pain and Pain Management Among University Students: Online Survey and Web-Based Education	Hong Kong
	Santiago, A. *et al.*^49^	Revista Gaúcha de Enfermagem	Automedicação em estudantes de enfermagem do Estado do Amazonas - Brasil	Brazil
	Virmani, S. *et al.*^50^	Clinical Epidemiology and Global Health	Antibiotic use among health science students in an Indian university: A cross-sectional study	India
	Rasheed, FA. *et al.*^51^	Journal of Pharmacy and Bioallied Sciences	Academic stress and prevalence of stress-related self-medication among undergraduate female students of health and non-health cluster colleges of a public sector University in Dammam, Saudi Arabia	Saudi Arabia
2016	Yadav, AK. *et al.*^52^	Journal of Nepal Medical Association	Self-prescription of paracetamol by undergraduate students in BP Koirala institution of health sciences	Nepal
	Williams, A. *et al.*^53^	Contemporary Nurse	Self-medication practices among undergraduate nursing and midwifery students in Australia: a cross-sectional study	Australia
	Johnson, D. *et al.*^54^	International Journal of Pharmacy and Pharmaceutical Sciences	Self-medication practice among medical, pharmacy and nursing students	India
	Iuras, A. *et al.*^55^	Revista Portuguesa de Estomatologia, Medicina Dentaria e Cirurgia Maxilofacial	Prevalence of self-medication among students of State University of Amazonas (Brazil)	Brazil
	Ali, AS. *et al.*^56^	Journal of the Pakistan Medical Association	Practices of self-medication with antibiotics among nursing students of institute of nursing, Dow University of Health Sciences, Karachi, Pakistan	Pakistan
2015	Mlinar, S. *et al.*^57^	Vojnosanitetski Pregled	Analysis of over-the-counter medicines use among nursing students	Slovenia
2014	Silva, FM da. *et al.*^58^	Revista Eletrônica de Enfermagem	Caracterização da prática de automedicação e fatores associados entre universitários do curso de Enfermagem	Brazil
	Martinez, JE. *et al.*^59^	Revista Brasileira de Reumatologia	Estudo da automedicação para dor musculoesquelética entre estudantes dos cursos de enfermagem e medicina da Pontifícia Universidade Católica - São Paulo	Brazil
2012	Santos, B dos. *et al.*^60^	Journal of the Health Science Institute	Incidência da automedicação em graduandos de Enfermagem	Brazil
2011	Jalapeña, B. *et al.*^61^	Revista Visión de Enfermería Actualizada	Automedicación en estudiantes de Enfermería	Argentina
	Souza, LAF. *et al.*^8^	Revista Latino-Americana de Enfermagem	The prevalence and characterization of self-medication for obtaining pain relief among undergraduate nursing students	Brazil
2007	Damasceno, DD. *et al.*^62^	Revista Mineira de Enfermagem	Automedicação entre graduandos de enfermagem, farmácia e odontologia da Universidade Federal de Alfenas	Brazil
2004	Magaldi, L. *et al.*^63^	Revista de La Faculdad de Medicina	Farmacovigilancia y hábitos de consumo de medicamentos en los estudiantes de la Escuela de Enfermería de la Universidad Central de Venezuela	Venezuela

**Table 3. Categorization:** of factors involved in the practice of self-medication among undergraduate nursing students

Categories and factors related to self-medication	Studies identifying the category/factor *n* (%)	
**Clinical conditions (*n*= 52)**	*n*	%
General pain (headaches, back pain, muscle pain) Flu, colds, and cough Gastrointestinal disorders Fever Sore throat Skin problems (allergies, dermatitis, abrasions) Dysmenorrhea Emotional and psychological problems Infections in general Urinary problems Insomnia Weakness and fatigue Parasitic infections Weight loss Sexual enhancement	52 42 24 22 18 17 14 8 5 4 3 3 2 1 1	100.0 80.7 46.2 42.3 34.6 32.7 26.9 15.4 9.6 7.7 5.8 5.8 3.9 1.9 1.9
**Reasons or motivations (*n*=54)**		
Financial/economic factors Lack of time for consultation Ease and speed of access to medication and/or pharmacies Previous experience with the illness and/or medication Using one's own knowledge of pharmacology Mild illnesses/symptoms Difficulty accessing healthcare services Long waiting times for appointments Quick resolution of severe symptoms Hostility from healthcare professionals Lack of resolution after a consultation Lack of trust in healthcare professionals Cultural factors	28 26 22 20 16 15 14 8 6 4 4 3 2	51.8 48.1 40.7 37.1 29.6 27.8 25.9 14.8 11.1 7.4 7.4 5.6 3.7
**Medication groups according to *Anatomical Therapeutic Chemical* (ATC) classification (*n*=54)**		
ATC J (systemic antibacterials or antifungals) ATC N (analgesics or opioids or antipyretics) ATC M (anti-inflammatories) ATC A (antiemetics or vitamins or minerals or antacids or laxatives) ATC R (antihistamines or decongestants or expectorants) ATC N (anxiolytics or antidepressants or sedatives or stimulants) ATC D (antifungals or antiseptics or antihistamines or anesthetics or topical antibiotics) ATC H (corticosteroids) ATC P (antiprotozoal or anthelmintic) ATC G (contraceptives)	33 33 27 15 14 8 6 2 2 1	61.1 61.1 50.0 27.8 25.9 14.8 11.1 3.7 3.7 1.9
**Recall period (*n*=22)**		
1-3 months 4-6 months 12 months 15 days	9 6 6 1	40.9 27.3 27.3 4.5
**Sources of advice (*n*=53)**		
Pharmacy attendants or pharmacists Fellow students Family members Previous medical prescriptions *Internet* Pharmacology and/or pathology books and/or package inserts and/or course materials Other non-medical professionals	37 31 29 13 12 6 6	69.8 58.5 54.7 24.5 22.6 11.3 11.3
**Perception of risks (*n*=47)**		
Adverse reactions and events Masking symptoms, leading to incomplete treatment and delayed/incorrect diagnoses. Risk of drug interactions Increase in antimicrobial resistance Considering the practice unsafe Leading to drug abuse and dependence Raising treatment and healthcare system costs Incorrect administration Intoxication	24 24 16 12 9 6 5 5 2	51.1 51.1 34.0 25.5 19.1 12.8 10.7 10.7 4.3
**Perception of benefits (*n*=21)**		
Promoting self-care Not overburdening the healthcare system Quickly resolving mild symptoms	17 4 2	80.9 19.1 9.5
**Knowledge (*n*=27)**		
Knowledge was insufficient about medications Knowledge was sufficient about the clinical condition and/or medication used Knowledge was sufficient only for certain drug classes Students in more advanced semesters had greater knowledge about medications Lack of exposure to the topic in pharmacology courses	10 10 8 3 1	37.0 37.0 29.6 11.1 3.7
**Beliefs and Attitudes (*n*=17)**		
Used to advise others on self-medication Believed antibiotics could be beneficial in casual situations Previous experiences with medications contributed to self-medication For simple and recurring illnesses, they believed there was no need to consult a doctor Self-medication conducted by qualified individuals is beneficial Did not take medications for severe illnesses Reading the package insert is important for self-medication Felt fear about misdiagnosis and drug effects	5 4 3 1 1 1 1 1	29.4 23.5 17.6 5.9 5.9 5.9 5.9 5.9
**Practices (*n*=20)**		
Frequent use of medications Combining different medications Continuous use of medication Stopping medication when symptoms disappear Being cautious about taking medication before or after meals Oral administration of medication Taking medication at the correct time Stopping medication in case of adverse reactions Using inappropriate medications for the clinical condition In earlier semesters, self-medication was practiced more frequently	11 4 3 2 1 1 1 1 1 1	55.0 20.0 15.0 10.0 5.0 5.0 5.0 5.0 5.0 5.0

*ATC: Anatomical Therapeutic Chemical (ATC) classification*

## Discussion

This scoping review provides a broad and detailed overview, covering the years 2004 to 2024, of self-medication among undergraduate nursing students. The findings^4^^-^^8^^,^^15^^-^^63^ showed that this behavior is generally influenced by clinical, socioeconomic, educational, and healthcare access factors.^4^^-^^8^^,^^15^^-^^63^

Almost all studies,^4^^-^^8^^,^^15^^-^^18^^,^^20^^,^^21^^,^^23^^-^^63^ regardless of country and year of publication, indicated that "general pain" and flu-like conditions are the main reasons for self-medication among undergraduate nursing students. These conditions are typically observed in young adults, ^4^^-^^8^^,^^15^^-^^18^^,^^20^^,^^21^^,^^23^^-^^63^ especially in academic environments where stress and exposure to pathogens are common; they are perceived as mild and self-limiting, which may lead to the belief that formal medical intervention is unnecessary.^34^^,^^43^ Additionally, this finding may be associated with the signs and symptoms experienced during the COVID-19 pandemic, as nearly half of the articles were published between 2020 and 2024, i.e., during or post-pandemic. 

Although most studies^4^^,^^6^^,^^7^^,^^15^^-^^29^^,^^31^^-^^41^^,^^43^^-^^50^^,^^52^^-^^62^ presented various risks of self-medication, there was widespread use of systemic antibiotics/antifungals (ATC J). This is an important finding that deserves attention, especially since these are nursing students, given the health recommendation for medical prescriptions to acquire this drug class, and the fact that there has been an international campaign for nearly a decade about AMR issues.^64^^,^^65^ This phenomenon represents a growing threat to global public health, where misuse, overuse, and lack of professional supervision are factors that tend to accelerate the process.^64^^,^^65^

Countries in Asia and South America were the main contributors to knowledge dissemination on the topic, suggesting that these regions have paid more attention to the problem of self-medication. However, these countries share certain aspects related to their healthcare systems,^66^^,^^68^ which may contribute to this behavior. The shortage of healthcare professionals, especially in remote and peripheral areas, combined with limited access to healthcare services and economic factors, are common elements that emerged in the students' self-reports. In these cases, the benefits of the practice were often associated with seeking self-care, corroborating previous research findings in the region.^69^^,^^70^

Advice^4^^-^^8^^,^^15^^-^^45^^,^^47^^-^^63^ for students came mainly from information provided by pharmacy professionals, not necessarily pharmacists, fellow students, followed by family members. In this sense, the evidence presented is concerning, requiring joint interpretation beyond understanding the practice itself and may provide support for potential interventions, especially in academic training.

Regarding knowledge, beliefs/attitudes, and practices about self-medication, the findings identified limited knowledge, negative beliefs/attitudes, and practices considered inappropriate/unsafe regarding the medications used.^4^^,^^6^^,^^16^^,^^23^^,^^27^^,^^30^^-^^32^^,^^34^^,^^36^^,^^38^^,^^39^^,^^41^^,^^42^^,^^44^^,^^45^^,^^47^^,^^49^^,^^50^^,^^52^^,^^53^^,^^55^^,^^59^ This set of findings requires attention and academic intervention in professional training. Nurses^71^ play a crucial role in the medication administration process, in preventing and recognizing ADEs. 

In this sense, the fragility in acquiring knowledge, especially in pharmacology during undergraduate studies, may represent one of the main causes involved in the occurrence of an ADE. It is essential to overcome certain barriers to incorporating knowledge, which may involve adjustments in the course's pedagogical project, teaching methods, and the need for theoretical-practical articulation about the benefits and risks of medications in different areas of student practice. Knowledge limited to classic aspects, such as 10 traditional certainties of the profession regarding drug preparation and administration, may contribute to the emergence of inadequate beliefs and practices that lead to significant risks.^72^^,^^73^

There were two limitations to consider in this scoping review. The recall period for self-medication was highly varied, which may underestimate or overestimate the practice; many studies included undergraduate nursing students and others from health sciences fields, which may have impacted the specific analysis for the nursing profession. 

Therefore, this review contributed to mapping the trend of self-medication. The identified evidence highlights the seriousness of this practice not only for students' health but also for patient safety. It seems essential to reflect critically and hold curricular discussions on the emerging need to include and incorporate content involving "safe use of medications" integrated into Pharmacology, Public Health, or Patient Safety courses in nursing curricula. Thus, it reinforces the development of competencies and skills involving this extensive topic during the teaching-learning process, especially in Asian and South American countries.

## Conclusion

This study showed that clinical, socioeconomic, and behavioral factors were related to self-medication among undergraduate nursing students, particularly in Asia and South America. Knowledge and beliefs/attitudes, which are conditioning elements of self-medication practice, despite being drivers of this behavior, were underexplored in the studies. 

It is emphasized that future nurses play a relevant role in the medication system in healthcare services since they are involved from preparation to monitoring outcomes after drug administration. It is essential that they develop good practices related to medication safety, which can substantially contribute to educating the population about the risks of adverse events resulting from unsafe medication use. 
